# Advisory warning labels: what do dietitians advise parents of food-allergic children?

**DOI:** 10.1186/2045-7022-3-S3-P127

**Published:** 2013-07-25

**Authors:** P Turner, A Fox

**Affiliations:** 1 Paediatric Allergy & Immunology, Imperial College London, London, UK; 2 Allergy Academy, Kings College London, London, UK

## Background

Advisory warning “may contain” labels (AWLs) are found on 70% of pre-packed foods, and significantly impact upon patients with food allergy and their families. We sought to assess the advice regarding avoidance of AWLs provided by dietitians involved in the management of food-allergic children.

## Methods

A questionnaire was developed to assess factors which might affect the advice provided by health professionals with regards to AWLs. Dietitians attending an allergy education day were invited to participate.

## Results

61 dietitians participated in the survey, 26% specialised in allergy management, 57% in paediatrics. 70% had been qualified for 5+ years.

38% mandated complete avoidance of foods labelled “may contain… nuts” (but with no nut listed in the ingredients) in nut-allergic individuals. 36% recommended avoidance of only specific foods (such as confectionery) while 12% advised that avoidance of foods with AWLs was not required so long as the child was free of intercurrent infection and/or other factors e.g. absence of recent exercise.

Factors which resulted in more stringent avoidance being recommended included: asthma (56% recommending complete avoidance, p<0.05); prior history of anaphylaxis (79%, p<0.01); prior mild reaction to a tiny amount (71%, p<0.05). Provision of an adrenaline auto-injector device did not affect the advice given (p>0.05). Where a nut-allergic child without a history of anaphylaxis was able to tolerate peanut, fewer dietitians recommended complete avoidance (20% vs 38%, p<0.05).

Advice did not vary significantly where the allergen in question was egg rather than a nut. Tolerance of baked egg (e.g. in a cake) did not affect the advice given. However, where there was a history of anaphylaxis to raw egg but tolerance to egg in baked foods, more dietitians advised avoidance of foods with an AWL to egg (43% vs 26%, p<0.05).

## Conclusion

Advice to avoid foods with AWLs was more widespread where there was a history of anaphylaxis, asthma or prior reaction to a tiny amount of allergen. Some of these recommendations contradict the available literature on risks of allergic reaction with consumption of pre-packed foods. We are currently surveying a wider population of health professionals to assess advice provided with regards to AWLs.

## Disclosure of interest

None declared.

## References

[B1] Turner PJ Kemp A Campbell DC Advisory food labels – time to provide the allergic consumer with more than just “traces” of useful information BMJ 2011 343 d6180 21998345 10.1136/bmj.d6180

